# Frost Resistance Number to Assess Freeze and Thaw Resistance of Non-Autoclaved Aerated Concretes Containing Ground Granulated Blast-Furnace Slag and Micro-Silica

**DOI:** 10.3390/ma12244151

**Published:** 2019-12-11

**Authors:** Eldar Sharafutdinov, Chang-Seon Shon, Dichuan Zhang, Chul-Woo Chung, Jong Kim, Saltanat Bagitova

**Affiliations:** 1 Department of Civil and Environmental Engineering, Nazarbayev University, Nur-Sultan 010000, Kazakhstan; esharafutdinov@nu.edu.kz (E.S.); jong.kim@nu.edu.kz (J.K.); 2 Department of Architectural Engineering, Pukyong National University, Yongso-ro 45, Nam-Gu Busan 48513, Korea; cwchung@pknu.ac.kr; 3 Department of Architecture and Civil Engineering, L.N. Gumilyov Eurasian National University, Nur-Sultan 010008, Kazakhstan; dinar_j@mail.ru

**Keywords:** frost resistance number, freeze-thaw resistance, non-autoclaved aerated concrete, ground granulated blast-furnace slag, micro-silica, the degree of saturation

## Abstract

Aerated concrete (AC), such as cellular concrete, autoclaved aerated concrete (AAC), and non-autoclaved aerated concrete (NAAC), having excellent insulation properties, is commonly used in buildings located in cold regions, such as Nur-Sultan in Kazakhstan, the second coldest capital city in the world, because it can contribute to a large energy saving. However, when the AC is directly exposed to the repeated freeze and thaw (F-T) cycles, its F-T resistance can be critical because of lower density and scaling resistance of the AC. Moreover, the evaluation of the F-T resistance of the AC based on the durability factor (DF) calculated by using the relative dynamic modulus of elasticity may overestimate the frost resistance of the AC due to the millions of evenly distributed air voids in spite of its weak scaling resistance. In the present study, the F-T resistance of NAAC mixtures with various binary or ternary combinations of ground granulated blast-furnace slag (GGBFS) and micro-silica was assessed mainly using the ASTM C 1262/C1262M-16 Standard Test Method for Evaluating the Freeze-Thaw Durability of Dry-Cast Segmental Retaining Wall Units and Related Concrete Units. Critical parameters to affect the F-T resistance performance of the NAAC mixture such as compressive strength, density, water absorption, air–void ratio (VR), moisture uptake, durability factor (DF), weight loss (W_loss_), the degree of saturation (S_d_), and residual strength (S_res_) were determined. Based on the determined parameter values, frost resistance number (FRN) has been developed to evaluate the F-T resistance of the NAAC mixture. Test results showed that all NAAC mixtures had good F-T resistance when they were evaluated with DF. Binary NAAC mixtures generally showed higher S_d_ and W_loss_ and lower DF and S_res_ than those of ternary NAAC mixtures. It was determined that the S_d_ was a key factor for the F-T resistance of NAAC mixtures. Finally, the developed FRN could be an appropriate tool to evaluate the F-T resistance of the NAAC mixture.

## 1. Introduction

Aerated concrete (AC) originated in Europe as one of the widely used types of lightweight concrete (LWC). Generally, the AC is made from cement, silica-rich materials, water, fine aggregates, and aluminum powder [1]. Aluminum powder reacts with alkalis in the cement and then forms millions of evenly distributed and uniformly sized small air bubbles in the concrete matrix required for the generation of the porous structure [2]. As any LWC, the AC, due to the unique highly porous structure, possesses better sound absorption caused by the converted air-borne sound energy in the minute channels of the concrete [3,4,5]. Moreover, the high porous characteristics of the AC give it excellent insulation properties contributing to a reduction in the energy consumption related to heating, ventilation, and air conditioning (HVAC) systems suited for severe environments [6,7,8]. However, the high prosody of AC also leads to reduced density and low compressive and flexural strengths compared to normal concrete. In order to overcome these shortcomings and obtain a good quantity of pores and evenly distributed pores, an autoclaved curing under high pressure and temperature is required [9].

Despite its excellent insulation property, the freeze and thaw (F-T) resistance of AC appears to be one of the most fundamental and emerging properties of AC when it is directly exposed to the outside environment [10]. Due to the high porosity of AC, a significant amount of freezable water existing within the pore structure (capillary and entrapped pores) subjected to the contact with the surface of the AC can be easily frozen and thawed. The repeated F-T cycles are responsible for continuous and disruptive internal pressure, which causes micro-cracking in the concrete and results in scaling and spalling [11,12]. Therefore, it is clear that a critical parameter contributing to F-T resistance of the AC is a degree of moisture saturation, which is the amount of moisture (absorbed free water) present inside or at the surface of the concrete structure. Moreover, it is essential to reduce the number of capillary pores in the concrete mixture where a significant amount of freezable water exits.

In addition, the durability factor (DF), calculated from the relative dynamic modulus of elasticity of the specimen, is frequently used to evaluate the F-T resistance of concrete. However, the DF may overestimate the frost resistance of the AC due to millions of evenly distributed air voids inside the AC [12]. For example, in spite of good DF in the AC, the AC sometimes suffers a large amount of weight loss because of its weak surface. Therefore, it is necessary to develop an appropriate tool to consider all parameters affecting the F-T resistance of the AC.

In the meantime, supplementary cementitious materials (SCM), such as ground granulated blast furnace slag (GGBFS), micro-silica (MS) called silica fume, and fly ash (FA), are often incorporated into the concrete to improve some physical properties and durability of concrete in aggressive environments [13,14]. For example, it has been well established that GGBFS, despite its relatively slow rate of reaction, is quite useful in producing concrete with low permeability and inducing significantly improved long-term age strength by converting the calcium hydroxide (CH) into calcium silicate hydrate (C-S-H). GGBFS also enhances the physical and chemical resistance of the concrete by reducing the number of capillary pores and the potential for ionic ingress, migration, and concentration [15,16]. However, controversy still exists for the use of GGBFS in concrete subjected to F-T cycles. For example, cold weather conditions limit the percentage of GGBFS that can be used in concrete due to potential retardation in setting and slow strength development depending on the alkali content in the concrete system [17,18].

Additionally, MS can induce close packing of materials, reduced bleeding, and reduced pore size, as well as generate more nucleation sites to accelerate hydration reactions because of its significant quantity of active silica and high specific surface area (typical size of 0.1–0.2 µm) [13,19,20]. The smaller size of the capillary pores in concrete containing MS decreases the total amount of freezable water, subsequently resulting in excellent resistance in F-T cycles. If GGBFS and MS are used together in AC, they may provide some synergistic effects. This would result in increasing the density, refining the pore structure, and reducing the permeability to make concrete less susceptible to F-T cycles along with appropriate air content.

From a sustainability point of view, GGBFS and MS are industrial by-products obtained by quenching molten iron slag from a blast furnace and silicon metal (ferrosilicon) produced in submerged electric arc furnaces, respectively. Both by-products are retreated and then commonly used in concrete to improve physical, chemical, mechanical, and durability performance by two primary mechanisms, such as a pozzolanic reaction and a micro-filler effect. Notably, both GGBFS and MS are produced in Karaganda, Kazakhstan, and used in the concrete of industrial and house buildings. Moreover, as stated previously, an autoclave curing is typically used for the AC, but it is not economical and is environmentally costly because of its high pressure and temperature operation. The authors’ previous work [21,22] indicated that 28-day compressive strength, porosity, and thermal conductivity of fully cured non-autoclaved aerated concrete (NAAC) is not much different from those of the autoclaved AC. Therefore, to develop a sustainable and durable NAAC mixture with the good F-T resistance and thermal energy conservation, GGBFS and MS were selected as the main materials to cast NAAC mixtures in this study.

In this research, NAAC mixtures with a ternary cementitious mixture system with varying proportions of Portland cement, GGBFS, and MS at the fixed amount of lime and aluminum powder have been explored from extensive laboratory experiments related to F-T resistance. Although a great deal of research associated with durability characterization of aerated concrete has been done, unfortunately, to date, there are few studies and insufficient data available which discuss and examine the F-T performance and critical parameters to affect the F-T resistance of the AC. Notably, there is no data available to evaluate the F-T resistance of the NAAC. Therefore, the F-T resistance of the NAAC was evaluated in terms of compressive strength, dry density, water absorption, air-void ratio, moisture uptake, and F-T resistance including durability factor, the degree of moisture saturation, weight loss, and residual strength. Based on these test results, a frost resistance number (FRN) has been developed to evaluate the F-T resistance of the NAAC mixture.

## 2. Experimental Program

### 2.1. Materials, Mixture Proportion, Mixing Procedure, and Casting Specimens

Materials used to cast NAAC in this study included silica-rich river sand from Red Flag pit (Aqmola, Kazakhstan), ASTM Type I Portland cement (Aktau, Kazakhstan), micro silica, GGBFS, lime, gypsum, aluminum powders, and water shown in Table 1. To obtain a uniform and constant gradation of aggregate, the amount of passing on each selected sieve was kept constant for oven-dried sand passing sieve No. 4 (4.72 mm).

To determine the mixture proportion which yields excellent physical properties and F-T resistance of NAAC, the combination of GGBFS and MS replacement levels by mass of cement was selected as 0%/5%/10%/15% and 0%/10%/15%/20% by mass of cement, respectively. All NAAC specimens were prepared at a water-to-cementitious material ratio (w/cm) of 0.50. Table 2 shows all sets of mixture proportions of NAAC and the amount of material required to fill the given volume regarding kg per 1 m^3^.

The NAAC preparation was carried out in the laboratory using a commercial pan-type mixer for a total time of 5 min. As there is no standard for NAAC preparation, the mixing was carried out by the following sequence: Dry materials, except aluminum (Al) powder, were placed into the mixer. After dry mixing for 30 s, 60% of the required amount of water was then introduced and mixed for 1 min. The required amount of Al powder, along with 50% of the remaining water amount was added to the mixer throughout 1 min. The obtained mix was allowed for 1 min-non-stirring resting while the mix residuals were collected from the mixer walls and beaters. Finally, the remaining amount of water was placed into the mixer and continued for 1 min and 30 s until a homogeneous CC mixture with no lumps was obtained.

**Figure 1:**
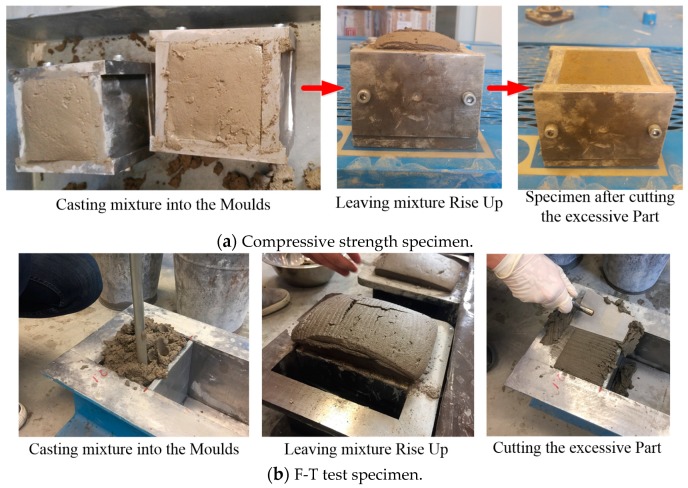
Casting compressive strength and F-T testing specimens.

As presented in Figure 1, cubical specimens with 70 mm × 70 mm × 70 mm dimensions were cast for compressive strength test while prismatic shape samples with 150 mm × 150 mm × 100 mm dimensions were cast for the F-T test. During the casting process, each specimen was cast in one layer with the compaction process performed manually with the help of a steel tapping rod. The increased surface part of the concrete specimen was manually removed using scrapers after 3 h of air-curing at ambient temperature. All specimens were covered with plastic in a room with 24 ± 2 °C for about 24 h. After 24-hour air-curing, the specimens were removed from molds and kept in a water tank at a temperature of 24 ± 2 °C until the time for compressive strength and F-T testing.

### 2.2. Test Procedures

#### 2.2.1. Determination of Void Ratio, Absorption, a Degree of Saturation of NAAC

Since the F-T resistance of the NAAC specimen is closely connected to the amount of freezable water inside the sample, the void ratio (open-cell air void), water absorption capacity, and the degree of saturation of NAAC mixtures were determined. The void ratio was calculated based on the difference between the oven-dry sample and the underwater-saturated sample using Equation (1) [23]:

(1) $$V_{r}=\left[1-\left(\frac{W_{\mathrm{sdw}}{-W}_{\mathrm{od}}}{\rho_{w}\times V}\right)\right]\times 100$$

where *V_r_* = void ratio (%); *W*_sdw_ = weight of saturated sample in water after immersion; *W*_od_ = weight of oven-dried sample in the air; ρ_w_ = density of water; and *V* = volume of sample.

According to the ASTM C 642 test method [24,25], the water absorption capacity of NAAC sample was determined using Equation (2):

(2) $$A=\frac{W_{\mathrm{sd}}{-W}_{\mathrm{od}}}{W_{\mathrm{sd}}}\times 100$$

where *A* = absorption after immersion in water (%); *W*_sd_ = weight of the surface-dry sample in air after immersion; and *W*_od_ = weight of the oven-dried sample in air.

The degree of saturation is defined as the weight of evaporable water contained in the specimen divided by the weight of evaporable water required for full saturation (from the oven-dry state to a constant weight state under vacuum saturation) [26] and calculated as:

(3) $$S_{d}=\frac{W_{n}{-W}_{\mathrm{od}}}{W_{s}{-W}_{\mathrm{od}}}\times 100$$

where *S*_d_ = the degree of saturation of the specimen; *W*_n_ = the weight of the in-test specimen; *W*_od_ = the weight after 24 h of oven drying at 100 ± 5 °C; and *W*_s_ = the weight after immersion to constant weight following saturation at 14-day water curing.

#### 2.2.2. Evaluation of F-T Resistance of NAAC

An F-T test was performed according to ASTM C 1262/C1262M-16 Standard Test Method for Evaluating the Freeze-Thaw Durability of Dry-Cast Segmental Retaining Wall Units and Related Concrete Units [27] with slightly modified procedures. The modified procedures include the measurement of (1) the relative dynamic modulus of elasticity during the F-T cycles instead of collecting the residue of the specimen at 20 cycles, (2) the weight change during F-T cycles, (3) the weight loss after 300 cycles (scaling of specimen), and (4) the residual strength of the samples before and after F-T test.

**Figure 2:**
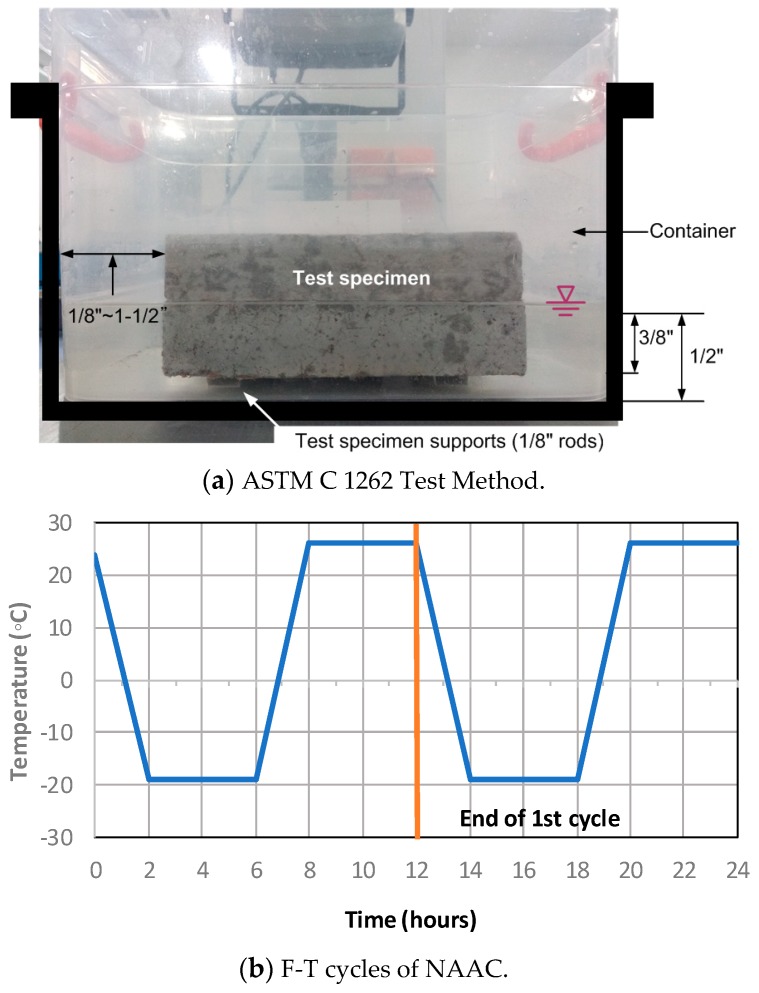
ASTM C 1262 test setup and F-T cycles of NAAC.

After 14-day water-saturated curing at 24 ± 2 °C, the prismatic sample of 150 mm × 150 mm × 100 mm size were oven-dried at 100 °C for at least 24 h. After cooling down to a temperature of 24 ± 2 °C, the specimen was weighed. As shown in Figure 2a, the specimen was placed into plastic containers filled with tap water onto plastic supports before proceeding to freeze-thawing cycles. Moreover, each sample was put into the separated container, and the water level was marked in order to control the consistency of the conducted experiment. The specimen was frozen in a temperature-humidity controlled environmental chamber for 4 h and thawed for 8 h (Figure 2b). Two F-T cycles were conducted per day, and total of 300 cycles were applied to the specimens (the C 1262/1262M indicates up to 50 cycles).

After 300 cycles, the amount of weight loss (an indication of a deterioration) was calculated by dividing the weight of residue by the dried weight of the specimen. The following equations give the weight of the residue and the weight loss of the specimen:

(4) $$W_{r}{=W}_{f+r}{-W}_{f}$$

(5) $$W_{\mathrm{loss}}=\frac{W_{r}}{W_{\mathrm{dried}}}\times 100$$

where *W*_r_ = weight of residue (spall); *W*_f+r_ = weight of the filter paper and dried residue; *W*_f_ = initial weight of the filter paper; *W*_loss_ = weight loss of the specimen (%); and *W*_dried_ = dried weight, respectively.

In order to evaluate the F-T resistance of the NAAC, the relative dynamic modulus of elasticity (RDME) of the specimens was calculated after conducting an ultrasonic pulse test using a Pundit Lab Ultrasonic Instrument (Zurich, Switzerland). Before each measurement, it was required to remove samples from containers, carefully get rid of surface water with a laboratory towel, weigh them on scales, calibrate the ultrasonic device, and measure the ultrasonic velocity and mass of specimens. The measurement was done only after to test specimens at every 10 cycles from 0 to 60 cycles and at every 30 cycles from 60 to 300 cycles. According to ASTM C260/C260M-10a (2016) Standard Specification for Air-Entraining Admixtures for Concrete [28], the test was continued for 300 cycles or until the RDME of the specimen had reached 60% of its initial value. The RDME was calculated by:

(6) $$P_{c}=\frac{v_{c}^{2}}{v_{0}^{2}}\times 100$$

where *P_c_* = the RDME at c cycles (as a percentage of the initial value, at 0 cycles); *v_c_* = the ultrasonic pulse velocity after c cycles of freezing and thawing; and *v_0_* = the ultrasonic pulse velocity at zero cycles of freezing and thawing [29].

The RDME at a given number of cycles is consequently used to calculate the durability factor (DF) of the NAAC samples. The DF of NAAC was checked after the completion of 300 F-T cycles using the following equation [30]:

(7) $$DF=\frac{PN}{300}$$

where *P* = RDME at N cycles (as a percentage of the initial value, at 0 cycles) and *N* = the number of cycles at which *P* falls below 60% (is taken to be 300 when remains higher than 60% after the completion of 300 cycles of F-T).

## 3. Experimental Results

### 3.1. Characteristics of Density, Void Ratio, Water Absorption, and Compressive Strength of NAAC

Generally speaking, dry density, void ratio, water absorption, and compressive strength of the NAAC are closely related to each other. With a decrease in density and an increase in the void ratio, the water absorption increases [12]. These properties play a critical role in the durability performance of the NAAC when it is mainly exposed to wet and F-T environmental conditions. If NAAC has a higher water absorption capacity, the NAAC mixture has a more chance to be damaged by the F-T cycle because the absorbed water inside the specimens can be easily frozen. Moreover, if the NAAC mixtures have the same air void content along with the even distribution in the NAAC matrix, the higher strength of the NAAC can provide better F-T resistance.

#### 3.1.1. Compressive Strength of NAAC

**Figure 3:**
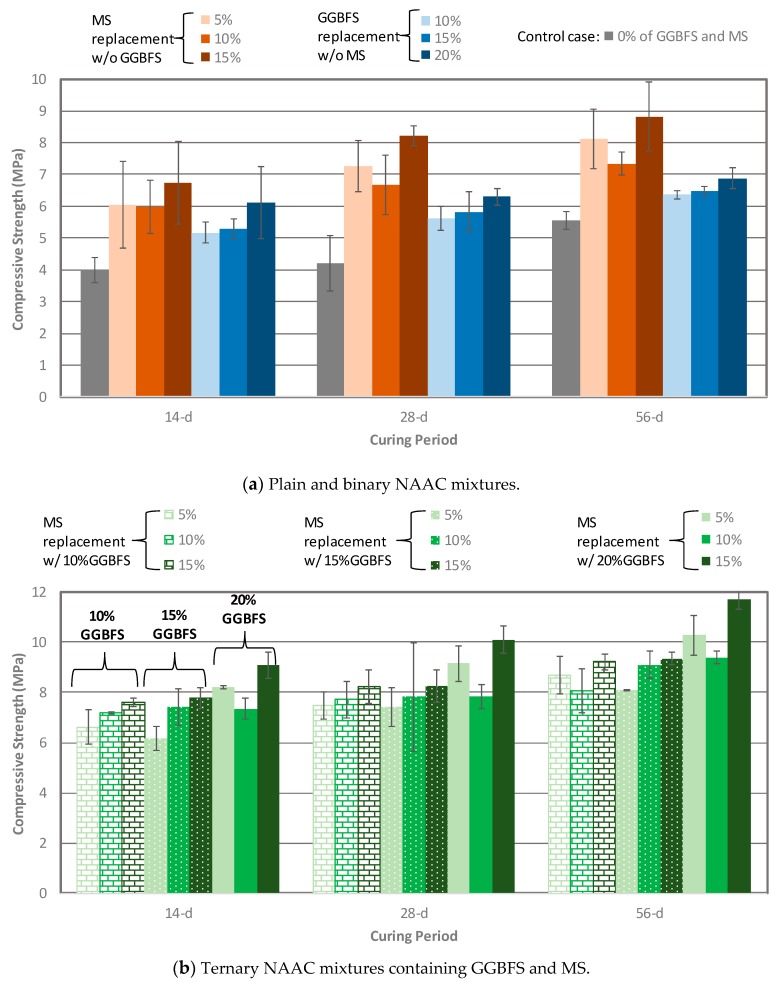
Compressive strength development of NAAC mixtures: (**a**) Plain and binary NAAC mixtures; (**b**) Ternary NAAC mixtures containing GGBFS and MS.

Compressive strength test were performed according to guidelines of ASTM C 109 [31] and The average of three specimens at the testing age was used for analysis. Figure 3 presents the compressive strength development of NAAC mixtures with various combinations of MS and GGBFS replacement percentages at 14-, 28-, and 56 days. The compressive strength of the NAAC increased over time for all mixtures regardless of binary and ternary mixtures. For binary NAAC mixture, the mixture containing SF had higher compressive strength than the GGBFS mixture and plain concrete. As previously stated, it is well known that the particle packing effect and the formation of additional C–S–H resulting from the pozzolanic reaction caused by SF would be responsible for increasing the strength [13]. When GGBFS is used in concrete, the degree of hydration of cement decreases due to the shortage of CH by replacing cement with GGBFS, eventually leading to decreasing the strength compared to the SF mixture [32]. In addition, compressive strength shows a similar trend of gradual increment along with a higher replacement level of SF and GGBFS except for mixture C-0GGBFS-10MS at curing age 28 and 56 days. The strength drop in mixture C-0GGBFS-10MS can be explained by the density to void ratio (D/VR) as shown in Table 3. The mixture with low compressive strength has a higher void ratio and lower density. This means that the lower the D/VR, the lower the compressive strength. Table 3 clearly shows that the mixture C-0GGBFS-10MS has D/*Vrs* of 33.84 and 36.29 at 28-days and 56-days, respectively.

Ternary mixtures show a similar strength trend with binary mixtures. When the amount of GGBFS was fixed, a higher MS replacement level yielded higher compressive strength except the mixtures C-10GGBFS-10MS at 56-day and C-20GGBFS-10MS at all ages. Like binary mixtures, the strength drops in these mixtures are related to the D/VR. It should be noted that these mixtures have lower D/VR values as shown in Table 3. Nevertheless, the observed trend justifies that the addition of SCMs such as GGBFS and MS to the NAAC mixture improves the compressive strength. The highest compressive strength has been achieved by the mixture C-20GGBFS-15MS regardless of curing time.

#### 3.1.2. Relationship between Compressive Strength, Void Ratio, and Dry Density

**Figure 4:**
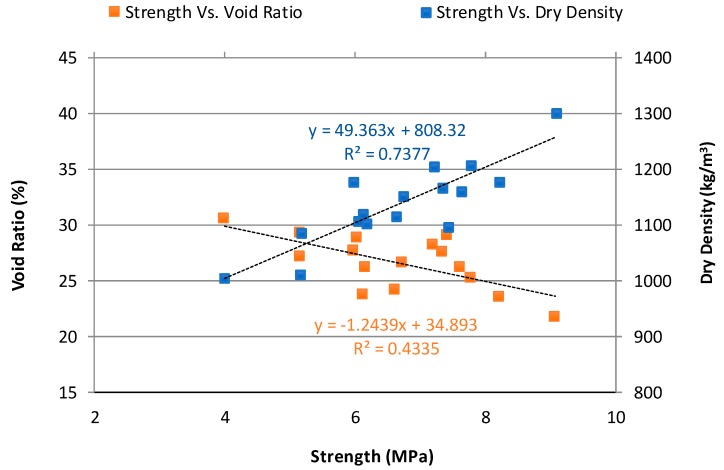
Relationship between compressive strength, void ratio, and dry density of NAAC mixtures.

The relationship between compressive strength, void ratio, and dry density of NAAC cured for 14-day before conducting the F-T test is plotted in Figure 4. The dry density and void ratio of 14-day cured NAAC samples ranges from 1005.6 kg/m^3^ and 30.6% for the plain mixture to 1302.0 kg/m^3^ and 21.7% for mixture C-20GGBFS-15MS, respectively. They depend on the combination of cementitious materials such as cement, GGBFS, and MS. As expected, the compressive strength generally increases with an increase in density, whereas NAAC mixtures with higher void ratio correlate with lower compressive strength.

#### 3.1.3. Relationship Between Water Absorption, Dry Density, and Void Ratio

**Figure 5:**
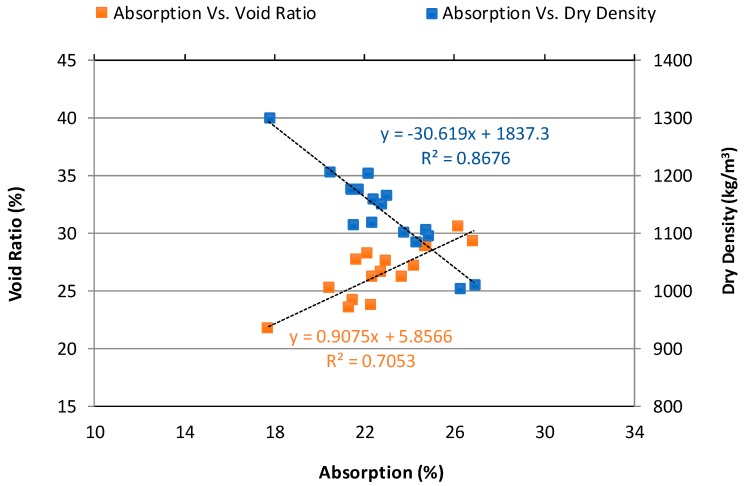
Relationship between absorption, void ratio, and dry density of NAAC mixtures.

Figure 5 shows the relationship between water absorption and dry density of NAAC cured for 14-day before conducting the F-T test. The mixtures with lower dry density absorb a higher percentage of water than those with higher density (a correlation coefficient is 86.76%). For instance, the plain concrete mixture with a dry density of 1005.6 kg/m3 and water absorption of 26.2% is supposed to be more vulnerable to F-T damage than mixture C-20GGBFS-15MS with a dry density of 1302.0 kg/m^3^ and water absorption of 17.7%. When these mixtures are exposed to wet and F-T cycles, the plain concrete tends to absorb much more water than the C-20GGBFS-15MS mixture. The absorbed water inside the mixture can be frozen and then eventually leads to poor F-T resistance.

The relationship between void ratio and water absorption of the NAAC is also presented in Figure 5. An increase in the void ratio of the NAAC increases in water absorption. However, a higher void ratio does not necessarily bring on higher water absorption as a linear regression coefficient between void ratio and water absorption was 70.53%. In fact, the void ratio of NAAC is the sum of all pores consisting of gel pores, capillary pores, other micropores produced a chemical reaction (e.g., a reaction of hydrated lime and aluminum powder), and entrapped air voids within the NAAC. It should be noted that the water absorption of the mixture is mainly related to capillary pores and open accessible pores and the connectivity of the pore system exposed to water (the water content of the saturated paste expressed as a percentage of its weight). Interestingly, these results match with the authors’ previous findings [12] using cellular concrete mainly made of a forming agent to form air-void structures, although the NAAC mixture creates the air-void systems by chemical reaction. From the relationship between water absorption, density, and void ratio of NAAC, it could be concluded that these parameters are closely related to each other, and the NAAC mixture with lower dry density results in an increasing void ratio and high-water absorption value.

### 3.2. Evaluation of Freeze-Thaw Resistance of NAAC

#### 3.2.1. Relative Dynamic Modulus of Elasticity (RDME)

**Figure 6:**
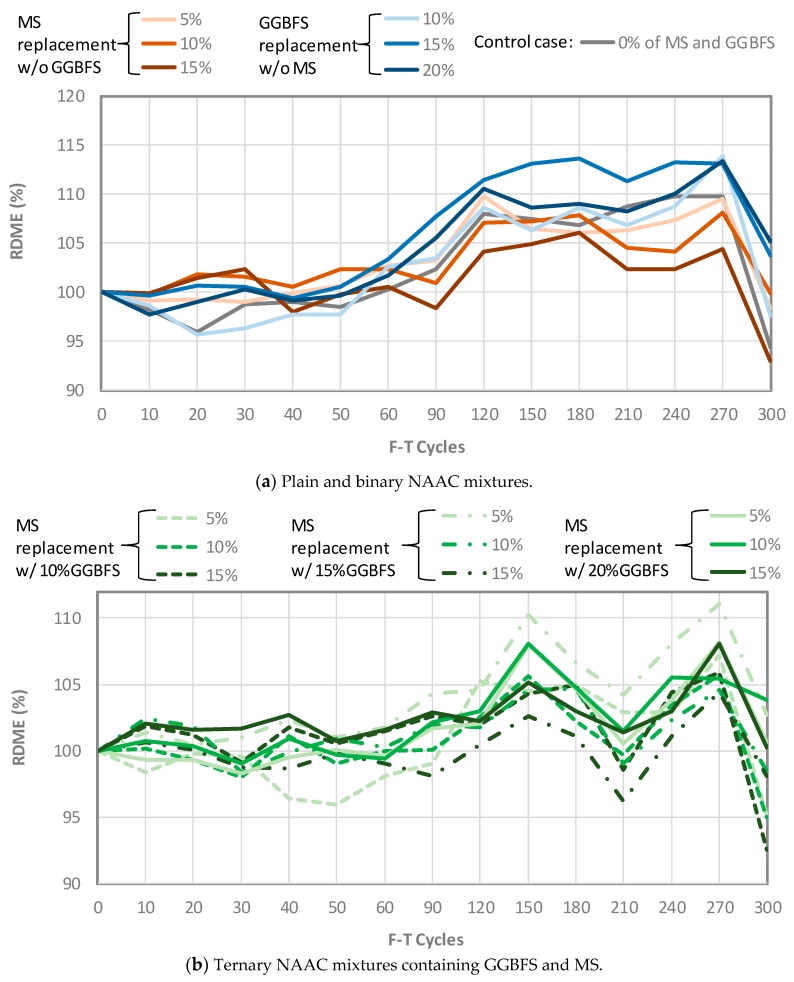
Relative dynamic modulus of elasticity of NAAC mixtures: (**a**) Plain and binary NAAC mixtures. (**b**) Ternary NAAC mixtures containing GGBFS and MS.

As shown in Figure 6, the F-T resistance of NAAC samples was evaluated based on the RDME. Generally, the RDME of concrete increases slightly due to cement hydration at initial stages, but the RDME of concrete suffering F-T damage decreases over time due to the development of internal microcracks damaged by F-T cycles. All mixtures including the plain and binary NAAC mixtures tend to sustain their durability properties during 300 F-T cycles, which indicates that RDME values of all mixtures do not show a significant drop from the initial 100% condition. The excellent F-T resistance of NAAC specimens might be attributed to improved air-void systems. The micro and macropores in the NAAC mixtures that were generated by chemical reaction seem to serve as reservoirs to reduce disruptive internal pressure built up by ice formation. Shon et al. [33] reported that air-voids less than 300 μm which are uniformly dispersed throughout the hydrated cement paste (hcp), play a critical role as reservoirs to absorb disruptive stresses caused by the formation of ice inside hcp, consequently preventing the damage due to F-T cycles. It shound be noted that the NAAC mixture contains millions of evenly distributed, uniformly-sized air bubbles or cells (up to 80% of the volume).

#### 3.2.2. Durability Factor (DF) of NAAC Mixture

**Figure 7:**
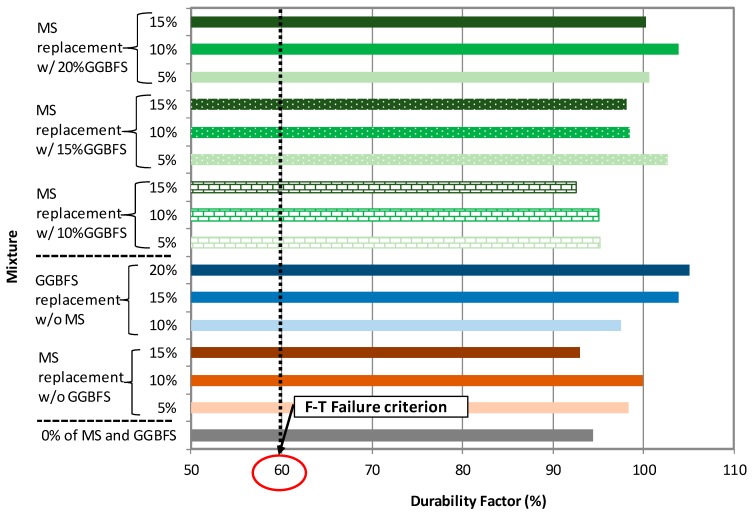
Durability Factor of each mixture after 300 F-T cycles.

The durability factor (DF) of all NAAC mixtures was obtained after the completion of 300 F-T cycles based on the corresponding RDME values using Equation (7) as illustrated in Figure 7. Like the RDME results, all mixtures possess DF values higher than 90%. This result indicates that all NAAC mixtures seem to develop suitable porous structure significantly contributing to the frost resistance because all NAAC mixtures meet the DF criterion that remains higher than 60% after the completion of 300 F-T cycles.

#### 3.2.3. Weight Change and Moisture Uptake of NAAC Mixtures

**Figure 8:**
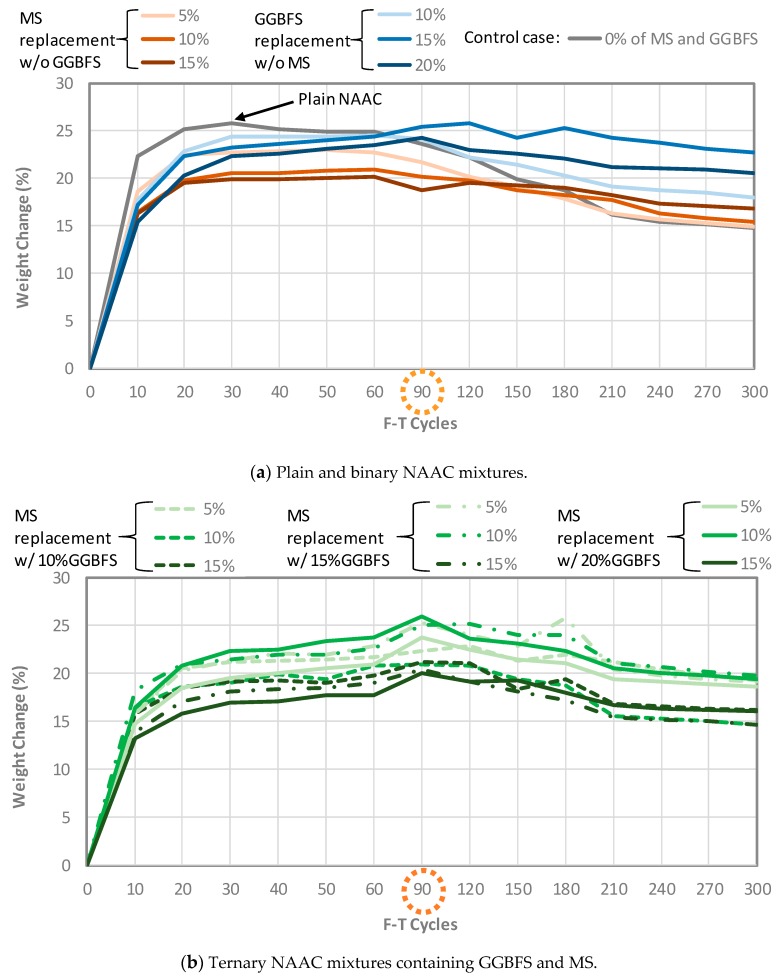
Weight changes of NAAC mixtures up to 300 F-T cycles: (**a**) Plain and binary NAAC mixtures; (**b**) Ternary NAAC mixtures containing GGBFS and MS.

Even though DF values of the NAAC mixtures remain above 90% after 300 F-T cycles, it is necessary to check the weight change of the NAAC mixture associated with scaling and spalling because the NAAC is a lightweight concrete and is more sensitive to the loss of weight. Figure 8 presents the percentage of weight change for all NAAC mixtures. All mixtures exhibited a sharp increase in mass induced by the water absorption up to an initial 10 cycles and a gradual increase in the mass to 90 F-T cycles, followed by a start in the reduction of their weights. It should be noted that all specimens were completely dried out before the F-T cycling test. As expected, the plain NAAC mixture reaches its highest weight reading during the first 30 cycles while other mixtures have their boundaries ending at cycles 60 and 90. The primary reasons for such a situation seem to be void ratio and absorption relationship with moisture uptake of NAAC samples.

**Figure 9:**
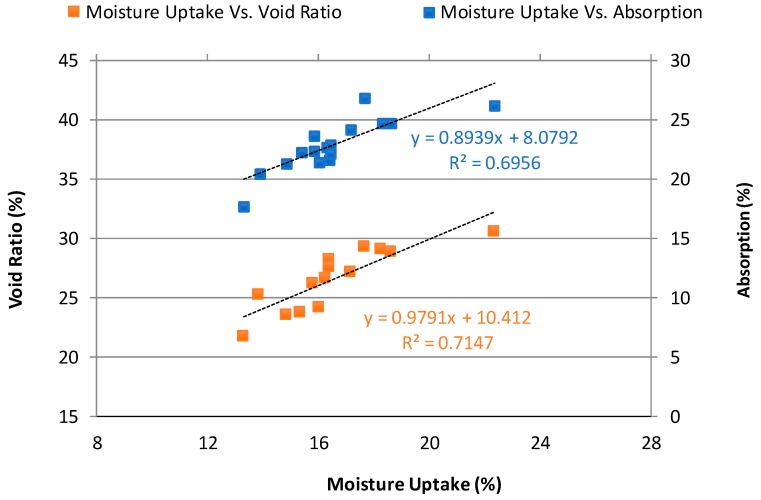
Relationship between moisture uptake, absorption, and void ratio of NAAC mixtures at 10 F-T cycles.

Figure 9 illustrates the relationship between moisture uptake, void ratio, and water absorption of NAAC mixtures at the First 10 F-T cycles. There is a direct dependency where the mixture with high values of water absorption and void ratio possess high moisture uptake readings as expected. Despite few discrepancies, this result confirms that the moisture uptake of concrete is primarily as a function of the void ratio regardless of the type of cementitious material with respect to the combination of GGBFS and MS [34].

## 4. Discussion and Frost Resistance Number

### 4.1. Factors to Influence the Performance of F-T Resistance of NAAC Mixtures

In cold climates, frost attack is a significant cause of damage to concrete unless adequate precautions are taken. The frost attack of concrete is classified into two types: (1) internal frost attack caused by freezing of moisture inside the concrete and (2) surface scaling caused by freezing of water in contact with the surface. Both types of attacks rely on how much moisture is present either inside the concrete or on the surface of the concrete. The moisture in the concrete associated with frost damage is free water in capillary and entrapped voids. The capillary porosity can be increased by increasing w/c. Increasing pore volume in such continuously-connected pore systems makes water flow channels increase, consequently leading to increased permeability that allows more water inside concrete to form ice [33]. Therefore, the susceptibility to frost attack is largely controlled by the amount of the capillary voids and entrapped voids that are connected to the degree of saturation (S_d_). The S_d_ is defined as the fraction of the air void system which has been filled with water. This means that the higher S_d_, the faster F-T damage starts.

**Figure 10:**
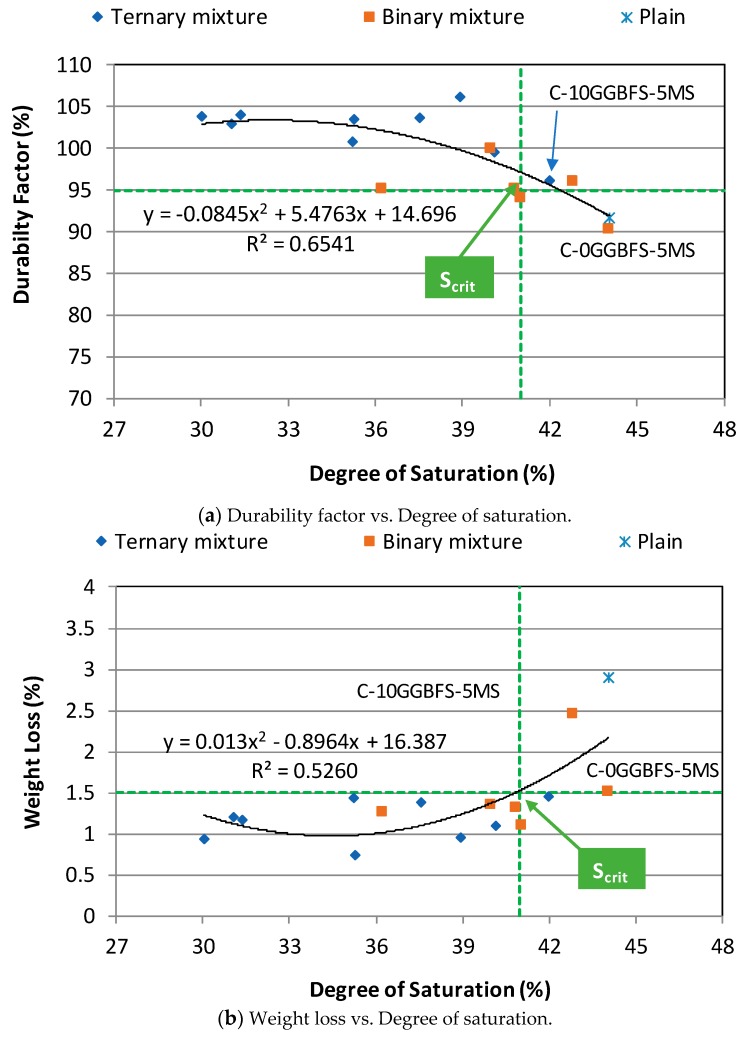

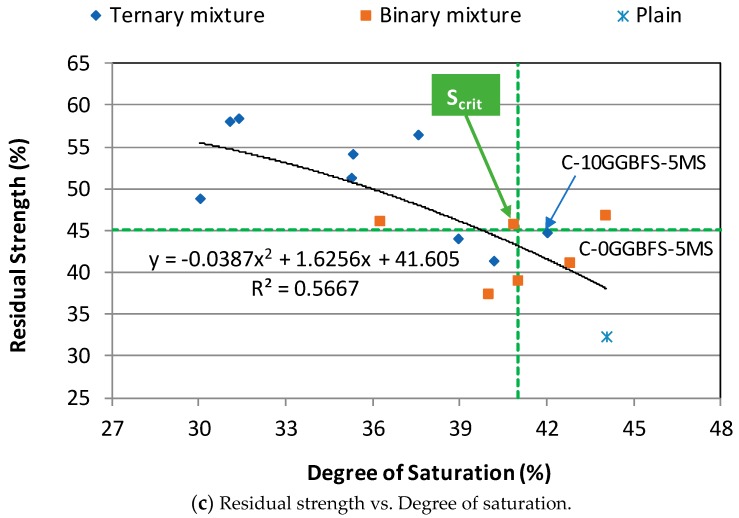
Relationship between durability factor, weight loss, residual strength, and degree of saturation of NAAC mixtures at 300 F-T cycles. (**a**) Durability factor vs. Degree of saturation; (**b**) Weight loss vs. Degree of saturation; (**c**) Residual strength vs. Degree of saturation.

Figure 10 illustrates DF, weight loss (W_loss_), and residual strength (S_res_) against the S_d_ after 300 F-T cycles. As expected, plain mixture C-0GGBFS-MS has the 2nd lowest DF of 91.7%, the highest W_loss_ of 2.91%, and the lowest S_res_ of 32.2% at the highest S_d_ of 44.05%. In spite of a little variation, ternary NAAC mixtures generally show lower S_d_ and W_loss_ and higher DF and S_res_ than those of binary NAAC mixtures. For instance, the ternary mixture C-10GGBFS-5MS has S_d_ of 42.0%, W_loss_ of 1.46%, DF of 96.0%, and S_res_ of 44.7%, whereas the binary mixture C-0GGBFS-5MS has S_d_ of 44.0%, W_loss_ of 1.53%, DF of 90.4%, and S_res_ of 41.1%. The repeated F-T cycles promote the destruction of the internal structure of NAAC mixture and lead to an increase in porosity and a decrease in bonding strength between hydrated cement paste and aggregate. Eventually, this induces the increase in the S_d_ [35,36]. It should be noted that the higher S_d_, the faster F-T damage starts, which results in increasing W_loss_ and decreasing S_res_.

The concept of S_d_ is further extended to a critical degree of saturation (S_crit_). When the concrete is exposed to any given FT cycle, there exists a critical value of the S_d_ beyond which the F-T damage of concrete can be initiated rapidly. This value is called S_crit_ and is defined as the maximum allowable fraction of the air-void system which has been filled with water. If the concrete has the lower S_d_ than the S_crit_, the concrete has no significant internal cracking and better F-T resistance even after a large number of F-T cycles. The determination of S_crit_ for binary and ternary NAAC mixtures from the relationship between DF, W_loss_, and S_res_ and S_d_ can be obtained from Figure 10. The value of S_crit_ was determined to be 41%. For example, the S_crit_ from the relationship between DF and S_d_ indicates the value of S_d_ when the DF of the concrete mixture drops below 95% as presented in Figure 10a. In the NAAC mixture, the threshold value of DF can become higher value due to high air void content having millions of evenly distributed and uniformly sized air bubbles although the DF for the concrete having the good F-T resistance is 60% after the completion of 300 cycles F-T cycles [27]. Moreover, the relationship between W_loss_ and S_d_ and S_res_ and S_d_ also supports that the S_crit_ of NAAC mixtures is 41%. Figure 10b,c clearly show that NAAC mixtures having the value of S_d_ higher than 41% have higher W_loss_ and lower S_res_.

Even if the air-void ratio is a key factor associated with the F-T resistance of the NAAC mixture, other parameters such as w/cm, compressive strength, and cement paste content containing other cementitious materials. Moreover, the F-T resistance of the NAAC at an early age is more strongly influenced by both W_loss_ and S_d_ than other concrete because of its air-void stability induced by volume expansion, slower hydration rate, relatively high capillary porosity, and higher absorption. From Figure 10, it was observed that these two factors strongly influenced the F-T resistance of the NAAC mixture.

### 4.2. Frost Resistance Number (FRN) to Evaluate F-T Resistance of NAAC Mixtures

The concept of frost resistance number (FRN) was first introduced by Gjorv et al. [37]. Parameters, such as air-void size in the range of 0–300 μm, w/c, cement paste content, and compressive strength, were used to develop the FRN because these parameters mainly influence on the F-T resistance of concrete. For example, if air-voids that are less than 300 μm are located closely with each other, they can absorb the pressure due to ice-formation, eventually leading to preventing the internal micro-cracking caused by repeated F-T cycles [33,38]. Moreover, Shon et al. [33] have modified Gjorv’s FRN to evaluate the concrete containing high volume ASTM class F fly ash. They have added the term of surface scaling resistance to the original FRN. However, since both FRNs that were proposed by Gjorv and Shon require measuring the volume of air-voids smaller than 300 μm, it seems not to be practical. It is not easy to determine air-voids that are smaller than 300 μm, which requires special tools. Therefore, the authors propose the new FRN to evaluate the F-T resistance of the NAAC.

As stated earlier, the DF of the NAAC mixture is always beyond 60% that is considered as a threshold value of the F-T damage due to its high air void contents consisting of millions of evenly distributed and uniformly sized air bubbles. It is difficult to use the DF as an evaluation criterion of the F-T resistance of the NAAC mixture. In order to evaluate the combined effect of all parameters on the F-T resistance of NAAC mixture, authors introduce the new concept of FRN which is expressed by the following equation:

(8) $$FRN=\left(\frac{VR}{p}\right)\times \left(\frac{1}{w/cm}\right)\times S_{\mathrm{res}}\times \left(\frac{100-W_{\mathrm{loss}}}{100}\right)\times \left(\frac{100-S_{d}}{100}\right)$$

where *FRN* = the frost resistance number; *VR* = air-void ratio; *p* = cement paste content of NAAC containing other cementitious materials; *w/cm* = water to cementitious material ratio; S_res_ = residual strength in MPa; W_loss_ = weight loss, and S_d_ = degree of saturation.

**Figure 11:**
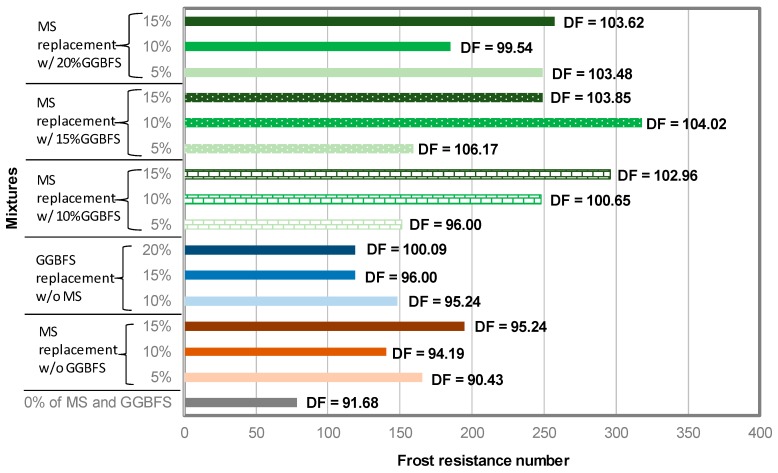
Frost resistance number of NAAC mixtures.

Figure 11 presents the FRN calculated using Equation (8) for each NAAC mixture. Plain and binary NAAC mixtures exhibited relatively lower FRN values than ternary mixtures, resulting in less 200. Interestingly, when plain mixture C-0GGBFS-0M which has DF of 91.68% is compared to the mixture C-0GGBFS-5MS with the DF of 90.43%, both mixtures have a similar value of the DF, but the plain mixture has two times smaller FRN than mixture C-0GGBFS-5MS. It should be noted that the plain mixture C-0GGBFS-0M showed higher absorption, S_d_, and W_loss_, and lower S_res_ compared to mixture C-0GGBFS-5MS. Therefore, the results of FRN to access the F-T resistance of NAAC mixture seem to be more reasonable than the DF.

## 5. Conclusions

The FRN to assess freeze and thaw resistance of non-autoclaved aerated concretes containing GGBFS and MS has been developed. The accomplishment of this research has been the comparison between compressive strength, dry density, void ratio, and water absorption capacity of NAAC mixtures and the determination of RDME, DF, weight change, moisture uptake, S_d_, W_loss_, and S_res_. Based on the test results, the following conclusions can be drawn:

(1) The mixtures with a higher MS replacement level yielded high compressive strength regardless of binary and ternary mixtures.
(2) While NAAC mixtures with high void ratio did not necessarily result in higher compressive strength, the density to void ratio dominates the compressive strength of NAAC mixtures (the lower the D/VR, the lower the compressive strength).
(3) Water absorption, dry density, and void ratio are closely related to each other, and the NAAC mixture with lower dry density results in increasing void ratio and high-water absorption value.
(4) All NAAC mixtures had good F-T resistance in terms of RDME and DF which presented more than 90%.
(5) The moisture uptake of the NAAC mixture is primarily a function of the void ratio regardless of the type of cementitious material concerning respect to the combination of GGBFS and MS.
(6) Binary NAAC mixtures generally show higher S_d_ and W_loss_ and lower DF and S_res_ than those of ternary NAAC mixtures. This means that higher S_d_, the faster F-T damage starts, which results in increasing W_loss_ and decreasing S_res_.
(7) Based on the relationship between DF, W_loss_, S_res_, and S_d_, S_crit_ of NAAC mixture was determined to 41%.
(8) The developed FRN seems to be an appropriate tool to evaluate the F-T resistance of the NAAC mixture.

Based on the findings in this study, ternary NAAC mixtures with the combination of GGBFS and MS show the better performance regarding the F-T resistance. Furthermore, the developed FRN shows the better accuracy than DF to evaluate the F-T resistance of NAAC. However, it should be noted that these findings are limited to the NAAC which is one type of aerated concretes. As previously stated, the AC also contains AAC and cellular concrete. In order to establish the newly proposed FRN to assess the F-T resistance of the AC, further testing must be conducted for different types of AC.

## Figures and Tables

**Table 1:** Chemical and physical properties of materials.

Chemical Compounds (wt %)	Cement	Microsilica	GGBFS	Aggregate	Physical Properties of Other Materials	
SiO_2_	18.20	98.00	34.20	68.90	Aggregate	-
Al_2_O_3_	4.01	0.45	11.90	9.03	Absorption	6.2%
Fe_2_O_3_	11.79	0.05	0.21	4.00	fineness modulus	1.02
Sum	34.00	97.5	46.31	81.83		
CaO	53.8	0.47	36.1	11.30	Gradation (total % passing)	
K_2_O	0.79	0.62	0.72	2.11	#4 (4.72 mm)	100
Na_2_O	0.16	-	0.73	1.69	#8 (2.36)	92.9
MgO	0.58	0.25	11.9	1.49	#16 (1.18 mm)	88.6
TiO_2_	0.20	-	1.75	0.49	#30 (0.60 mm)	85.2
MnO	0.46	-	0.51	0.26	#50 (0.30 mm)	82.6
P_2_O_5_	-	-	-	0.23	#100 (0.15 mm)	49.2
SO_3_	4.32	0.12	2.12	-	#200 (0.075 mm)	6.9
Free CaO	-	-	-	-	Pan	0.0
L.O.I	-	-	-	-	Lime (SG)	2.20
Fineness	^1^ 32.38	^2^ 48.4	^2^ 10.8	-	Gypsum (SG)	2.30
^3^ SG	3.14	2.22	2.90	2.40	Al powder (SG)	2.70

^1^ Amount retained on the no. 325 sieve; ^2^ Particle size distribution (median value, µm); ^3^ Specific gravity.

**Table 2:** Mixture proportions of NAAC.

Mixture	Unit Weight (kg/m^3^)							
	Sand	Cement	MS	GGBFS	Lime	Gypsum	Water	Al Power
C-0GGBFS-0MS	1091.5	471.0	0.0	0.0	110.0	58.0	319.5	1.9
C-0GGBFS-5MS	1099.8	447.5	16.7	0.0	110.0	58.0	316.1	1.9
C-0GGBFS-10MS	1108.1	423.9	33.3	0.0	110.0	58.0	312.6	1.9
C-0GGBFS-15MS	1116.4	400.4	50.0	0.0	110.0	58.0	309.2	1.9
C-10GGBFS-0MS	1095.8	423.9	0.0	43.5	110.0	58.0	317.7	1.9
C-15GGBFS-0MS	1098.0	400.4	0.0	65.3	110.0	58.0	316.8	1.9
C-20GGBFS-0MS	1100.2	376.8	0.0	87.0	110.0	-	315.9	1.9
C-10GGBFS-5MS	1104.1	400.4	16.7	43.5	110.0	58.0	314.3	1.9
C-10GGBFS-10MS	1112.4	376.8	33.3	43.5	110.0	58.0	310.8	1.9
C-10GGBFS-15MS	1120.7	353.3	50.0	43.5	110.0	58.0	307.4	1.9
C-15GGBFS-5MS	1106.3	376.8	16.7	65.3	110.0	58.0	313.4	1.9
C-15GGBFS-10MS	1114.6	353.3	33.3	65.3	110.0	58.0	309.9	1.9
C-15GGBFS-15MS	1122.8	329.7	50.0	65.3	110.0	58.0	306.5	1.9
C-20GGBFS-5MS	1108.4	353.3	16.7	87.0	110.0	58.0	312.5	1.9
C-20GGBFS-10MS	1116.7	329.7	33.3	87.0	110.0	58.0	309.0	1.9
C-20GGBFS-15MS	1125.0	306.2	50.0	87.0	110.0	58.0	305.6	1.9

Note: C-aGGBFS-bMS: C = cement; aGGBFS = % of GGBFS in cementitious materials; bMS = % of micro-silica in cementitious materials.

**Table 3:** Density and void ratio test results of NAAC.

Mixture	Density (D)			Void Ratio (*V_r_*)			Density to Void Ratio (D/VR)		
	14	28	56	14	28	56	14	28	56
C-0GGBFS-0MS	1774.8	1762.5	1768.2	43.0	51.6	51.9	32.9	34.2	34.1
C-0GGBFS-5MS	1781.8	1828.3	1834.9	44.8	47.0	44.2	38.5	38.9	41.5
C-0GGBFS-10MS	1751.7	1762.6	1763.6	50.8	52.1	48.6	42.5	33.8	36.3
C-0GGBFS-15MS	1828.5	1838.7	1817.7	45.4	48.7	43.0	43.4	37.8	42.3
C-10GGBFS-0MS	1784.0	1783.3	1784.4	50.5	49.6	48.8	34.6	36.0	36.6
C-15GGBFS-0MS	1777.7	1789.0	1786.4	50.2	50.4	47.1	40.0	35.5	37.9
C-20GGBFS-0MS	1782.3	1784.2	1780.9	51.3	50.6	49.1	47.2	35.2	36.3
C-10GGBFS-5MS	1759.7	1728.9	1776.6	49.9	45.6	49.8	46.1	37.9	35.7
C-10GGBFS-10MS	1778.0	1748.4	1779.9	51.0	47.2	50.4	42.7	37.0	35.3
C-10GGBFS-15MS	1804.0	1781.5	1812.7	48.8	45.6	46.5	44.4	39.1	39.0
C-15GGBFS-5MS	1775.3	1778.8	1786.6	49.8	49.7	50.1	42.2	35.8	35.7
C-15GGBFS-10MS	1802.3	1807.6	1785.8	48.3	49.2	45.6	37.7	36.8	39.2
C-15GGBFS-15MS	1788.6	1803.0	1786.6	48.8	47.7	49.0	47.9	37.8	36.5
C-20GGBFS-5MS	1822.8	1865.1	1827.5	45.2	47.1	46.7	50.1	39.6	39.2
C-20GGBFS-10MS	1820.0	1836.2	1803.9	45.2	47.8	46.1	42.3	38.4	39.2
C-20GGBFS-15MS	1850.0	1861.4	1854.4	40.4	45.6	45.9	60.0	40.8	40.4

## References

[1] Kurama H., Topcu I.B., Karakurt C. (2009). Properties of the autoclaved aerated concrete produced from coal bottom ash. J. Mater. Process. Technol..

[2] Chaipanich A., Chindaprasirt P. (2015). The properties and durability of autoclaved aerated concrete masonry blocks. Eco-Efficient Masonry Bricks and Blocks: Design, Properties and Durability.

[3] Narayanan N., Ramamurthy K. (2000). Structure and properties of aerated concrete: A review. Cem. Concr. Compos..

[4] Van Rooyen A.S. (2013). Structural Lightweight Aerated Concrete. Master’s Thesis.

[5] Zhu H., Wan K.T., Satekenova E., Zhang D., Leung C., Kim J. (2018). Development of lightweight strain hardening cementitious composite for structural retrofit and energy efficiency improvement of unreinforced masonry housings. Constr. Build. Mater..

[6] Narayanan N., Ramamurthy K. (2000). Microstructural investigations on aerated concrete. Cem. Concr. Res..

[7] Jones M.R., McCarthy A. (2006). Relations between structure and mechanical properties of autoclaved aerated concrete. Cem. Concr. Res..

[8] Ulykbanov A., Sharafutdinov E., Chung C.W., Zhang D., Shon C.S. (2019). Performance-based Model to Predict Thermal Conductivity of Non-Autoclaved Aerated Concrete through Linearization Approach. Constr. Build. Mater..

[9] Alexanderson J. (1979). Heat of hydration in foamed concrete: Effect of mix constituents and plastic density. Cem. Concr. Res..

[10] U.S. Department of Transportation (2006). Freeze-Thaw Resistance of Concrete with Marginal Air Content.

[11] Penttala V. (2006). Surface and internal deterioration of concrete due to saline and non-saline freeze-thaw loads. Cem. Concr. Res..

[12] Shon C.-S., Lee D., Kim J.H., Chung C.-W. (2018). Freezing and thawing resistance of cellular concrete containing binary and ternary cementitious mixtures. Constr. Build. Mater..

[13] Chung C.-W., Shon C.-S., Kim Y.S. (2010). Chloride ion diffusivity of fly ash and silica fume concretes exposed to freeze-thaw cycles. Constr. Build. Mater..

[14] Chen Y.L., Ko M.S., Chang J.E., Lin C.T. (2018). Recycling of desulfurization slag for the production of autoclaved aerated concrete. Constr. Build. Mater..

[15] Mehta P.K., Monteiro P.J.M. (1993). Concrete Structure, Properties, and Materials.

[16] Mindess S., Young J.F., Darwin D. (2003). Concrete.

[17] Wawrzeńczyk J., Juszczak T., Molendowska A. (2016). Determining equivalent performance for frost durability of concrete containing different amounts of ground granulated blast furnace slag. Bull. Pol. Acad. Sci. Tech. Sci..

[18] Zhang S.Y., Fan Y.F., Wzng Q. (2015). Experiment study on the effect of GGBFS on frost resistance of concrete. Key Eng. Mater..

[19] Folagbade S.O. (2012). Effect of fly ash and silica fume on the sorptivity of concrete. Int. J. Eng. Sci. Technol..

[20] Bektimirova U., Shon C.S., Zhang D., Sharafutdinov E., Kim J.R. (2018). Proportioning and Characterization of Reactive Powder Concrete for an Energy Storage Pile Application. Appl. Sci..

[21] Ulykbanov A., Sharafutdinov E., Ramazanova E., Shon C.-S. (2018). Thermal conductivity of aerated concrete based on response surface method. Mater. Sci. For..

[22] Sharafutdinov E., Abdigaliyev A., Sheriyev A., Zhang D., Shon C.-S. (2018). Properties of non-autoclaved aerated concrete with quadruple cementitious mixture using Response Surface Method. Mater. Sci. For..

[23] Schaefer V.R., Wang K., Suleiman M.T., Kevern J.T. (2006). Mix Design Development for Pervious Concrete in Cold Weather Climates.

[24] American Society for Testing and Materials (2013). ASTM C642-13 Standard Test Method for Density, Absorption, and Voids in Hardened Concrete.

[25] Foraboschi P., Vanin A. (2015). Mechanical behavior of the timber-terrazzo composite floor. Constr. Build. Mater..

[26] Li W., Pour-Ghaz M., Castro J., Weiss J. (2012). Water absorption and critical degree of saturation relating to freeze-thaw damage in concrete pavement joints. J. Mater. Civ. Eng..

[27] American Society for Testing and Materials (2016). ASTM C1262/C1262M-16 Standard Test Method for Evaluating the Freeze-Thaw Durability of Dry-Cast Segmental Retaining Wall Units and Related Concrete Units.

[28] American Society for Testing and Materials (2016). ASTM C260/C260M-10a (2016) Standard Specification for Air-Entraining Admixtures for Concrete.

[29] Sabir B.B. (1997). Mechanical properties and frost resistance of silica fume concrete. Cem. Concr. Comp..

[30] American Society for Testing and Materials (2015). ASTM C666/C666M-15 (2015) Standard Test Method for Resistance of Concrete to Rapid Freezing and Thawing.

[31] American Society for Testing and Materials (2016). ASTM C109 Standard Test Method for Compressive Strength of Hydraulic Cement Mortars.

[32] Lee H.S., Wang X.Y., Zhang L.N., Koh K.T. (2015). Analysis of the optimum usage of slag for the compressive strength of concrete. Materials.

[33] Shon C.-S., Abdigaliyev A., Bagitova B., Chung C.-W., Kim D. (2018). Determination of air-void system and modified frost resistance number for freeze-thaw resistance evaluation of ternary blended concrete made of ordinary Portland cement/silica fume/class F fly ash. Cold Reg. Sci. Technol..

[34] Kearsley E.P., Wainwright P.J. (2001). Porosity and permeability of foamed concrete. Cem. Concr. Res..

[35] You L., You Z., Dai Q., Guo S., Wang J., Schultz M. (2018). Characteristics of water-foamed asphalt mixture under multiple freeze-thaw cycles: Laboratory evaluation. J. Mater. Civ. Eng..

[36] Khanfour M.-K., Refai A.E. (2017). Effect of freeze-thaw cycles on concrete reinforced with basalt-fiber reinforced polymers (BFRP) bars. Constr. Build. Mater..

[37] Gjorv O.E., Okkenhaug R., Bathen E., Husevag R. (1978). Frost resistance and air-void characteristics in hardened concrete. Nord. Concr. Res..

[38] Pigeon M., Pleau R. (1995). Durability of Concrete in Cold Climates.

